# National governance of de-implementation of low-value care: a qualitative study in Sweden

**DOI:** 10.1186/s12961-022-00895-2

**Published:** 2022-09-01

**Authors:** Hanna Augustsson, Belén Casales Morici, Henna Hasson, Ulrica von Thiele Schwarz, Sara Korlén Schalling, Sara Ingvarsson, Hanna Wijk, Marta Roczniewska, Per Nilsen

**Affiliations:** 1 Procome Research Group, Department of Learning, Informatics, Management and Ethics, Medical Management Centre, Karolinska Institutet, Stockholm, Sweden; 2 Unit for Implementation and Evaluation, Center for Epidemiology and Community Medicine (CES), Stockholm, Sweden; 3 Department of Business Studies, Uppsala University, Uppsala, Sweden; 4 School of Health, Care and Social Welfare, Mälardalen University, Västerås, Sweden; 5 Division of Public Health, Department of Health, Medical and Caring Sciences, Linköping University, Linköping, Sweden

**Keywords:** Overuse, Low-value care, Disinvestment, De-implementation, Healthcare governance

## Abstract

**Background:**

The de-implementation of low-value care (LVC) is important to improving patient and population health, minimizing patient harm and reducing resource waste. However, there is limited knowledge about how the de-implementation of LVC is governed and what challenges might be involved. In this study, we aimed to (1) identify key stakeholders’ activities in relation to de-implementing LVC in Sweden at the national governance level and (2) identify challenges involved in the national governance of the de-implementation of LVC.

**Methods:**

We used a purposeful sampling strategy to identify stakeholders in Sweden having a potential role in governing the de-implementation of LVC at a national level. Twelve informants from nine stakeholder agencies/organizations were recruited using snowball sampling. Semi-structured interviews were conducted, transcribed and analysed using inductive thematic analysis.

**Results:**

Four potential activities for governing the de-implementation of LVC at a national level were identified: recommendations, health technology assessment, control over pharmaceutical products and a national system for knowledge management. Challenges involved included various vested interests that result in the maintenance of LVC and a low overall priority of working with the de-implementation of LVC compared with the implementation of new evidence. Ambiguous evidence made it difficult to clearly determine whether a practice was LVC. Unclear roles, where none of the stakeholders perceived that they had a formal mandate to govern the de-implementation of LVC, further contributed to the challenges involved in governing that de-implementation.

**Conclusions:**

Various activities were performed to govern the de-implementation of LVC at a national level in Sweden; however, these were limited and had a lower priority relative to the implementation of new methods. Challenges involved relate to unfavourable change incentives, ambiguous evidence, and unclear roles to govern the de-implementation of LVC. Addressing these challenges could make the national-level governance of de-implementation more systematic and thereby help create favourable conditions for reducing LVC in healthcare.

**Supplementary Information:**

The online version contains supplementary material available at 10.1186/s12961-022-00895-2.

## Background

The importance of providing evidence-based healthcare is widely recognized. This includes efforts to de-implement—that is, to reduce or stop the use of methods that are not evidence-based. These are usually referred to as low-value care (LVC), which is “care that is unlikely to benefit the patient given the harms, cost, available alternatives, or preferences of the patient” [1]. It has been estimated that 10–30% of all healthcare methods have little or no benefit to the patient [2] and that 12–15% of patients receive at least one LVC practice a year [3]. Almost three quarters of physicians in the United States report that they typically prescribe unnecessary tests or procedures at least once a week [4]. The prevalence of LVC in Europe has not been quantified but is highlighted as a considerable problem for healthcare systems [5]. In Sweden, LVC is listed in the national guidelines as do-not-do recommendations, and an investigation of the prevalence of these practices showed that only one of six practices decreased after being labelled as do-not-do [6].

De-implementing LVC in healthcare is important to improving patient and population health, maintaining public trust, minimizing patient harm and reducing unnecessary resource waste in healthcare and public health [7]. The increasing costs of new healthcare technologies and treatments in combination with increasing needs associated with ageing populations further underline the necessity of using resources wisely [8]. Thus, an important issue for researchers and policy-makers is in recognizing factors affecting LVC use and improving knowledge regarding the strategies that could be effective for reducing LVC. A recent study identified key factors on the national level influencing the use of LVC, which were found to be similar across three countries (United States, Canada, Netherlands) despite differences in their health systems [9]. The factors included the provider payment system, the pharmaceutical and medical device industry, fear of malpractice litigation, biased evidence and knowledge, medical education, and social factors around healthcare including medical and public culture. A previous study on de-implementation of LVC in primary healthcare in Sweden found that physicians’ use of LVC was influenced by uncertainty and disagreement about what not to do, perceived pressure from others in the healthcare system, and the desire to do something for the patients [10]. Thus, clinicians are influenced both by individual factors and by factors in the wider healthcare system to provide LVC, which highlights the importance of working strategically at different levels in the healthcare system to reduce LVC [7, 11]. This is supported in a recent Swedish study which found that both local strategies and systemic strategies are likely required to de-implement LVC in primary healthcare in Sweden [12].

One area that has received limited research interest is health system governance for de-implementing LVC. Health system governance includes the means to fulfil the objectives of healthcare systems [13] and is manifested in multiple forms, such as in legally binding laws and regulations, advisory guidelines (soft governance) [14], terms for priority-setting and resource allocation, and systems for performance-monitoring and accountability [15].

Governance of healthcare systems is inherently complex [15]. One reason is that it involves a wide range of stakeholders at multiple system levels, which commonly results in the coexistence of national-level guidelines and policies [16] in parallel with regional- and local-level decision-making and guidelines.

A scoping review by Nilsen et al. [17] identified four frameworks that addressed determinants of the de-implementation of LVC. Most frameworks focused on micro- and meso-level determinants such as healthcare professionals’ attitudes and beliefs, patient experiences and the immediate practice environment. Only one of the frameworks [18] accounted for governance in relation to policy, management and/or clinical decision-making at the meso (institutional) level. Another scoping review [11] identified nine empirical studies that examined determinants for de-implementing LVC, of which only three addressed governance in the form of “policy and political support” for the de-implementation of LVC.

Despite governance being identified as a determinant for de-implementation, there is limited knowledge about how the de-implementation of LVC is governed and what challenges might be involved in the governance of de-implementation. Addressing these knowledge gaps, we aimed in this study to explore national-level governance of the de-implementation of LVC in Sweden. The purpose is twofold: (1) to identify key stakeholders’ activities in relation to de-implementing LVC in Sweden at the national governance level and (2) to identify challenges involved in the national governance of the de-implementation of LVC in Swedish healthcare.

## Methods

### Design

The study was a qualitative interview study using thematic analysis [19]. The Standards for Reporting Qualitative Research were used to report the methods [20] (Additional file 1).

### Setting

The Swedish healthcare system, where the current study is set, provides a relevant empirical example of the governance and de-implementation of LVC, as both the use of scientific evidence and striving for cost-efficiency are core objectives of the Swedish healthcare system. These objectives are reflected in national and regional governance strategy [15, 21]. Sweden also has a long tradition of producing and using evidence to guide practice, involving a broad range of national-level stakeholders.

Sweden has a publicly funded (84%) and highly decentralized healthcare system, with considerable autonomy for the 21 regions (previously known as county councils) responsible for funding and providing healthcare to its citizens. Both public and private providers operate in the publicly funded healthcare market. Private providers are more common in primary care, in particular in urban areas [21]. At the national level, governance is performed by the Ministry of Health and Social Affairs, which sets overall goals and issues policies, as well as through several national public agencies responsible for supporting the implementation of policies issued by the ministry. Thereby, the national-level governance sets the frame for the regional governance of healthcare. This study focuses on the activities conducted and the challenges involved in governing de-implementation of LVC at the national level.

### Recruitment procedure and participants

We used a purposeful sampling strategy to identify national-level stakeholders based on the criteria of having a potential role in the governance and de-implementation of LVC based on their functions. We identified stakeholders based on recent governmental investigations into knowledge governance, emphasizing the role of government and nongovernmental agencies [22], and more general reviews of the governance of the Swedish healthcare system [15, 21]. The list of relevant stakeholders to invite to the study consisted of eight government agencies. In addition to these, the Swedish Association of Local Authorities and Regions (SALAR) which represents the regional and municipal governments, was included. The reason for including SALAR despite it not being a national-level actor was because of its leading and coordinating role in the national collaboration for knowledge governance (Table 1).

**Table 1:** Identified stakeholders and their functions of relevance for the de-implementation of LVC at a national level

Stakeholders	Function	Number of informants
Swedish Agency for Health Technology Assessment and Assessment of Social Services	Assesses the evidence for methods of health and social care based on systematic literature reviews	2
National Board of Health and Welfare (NBHW)	Provides soft law recommendations, e.g. develops national guidelines, based on the state of the evidence and professional expertise, to guide regional decision-makers and provider organizations in making priorities and organizing healthcare provision	1
Medical Products Agency	Makes direct decisions concerning medical products. Approves pharmaceuticals to access the Swedish market and provides evidence-based guidelines for the prescription and use of pharmaceuticals	2
Dental and Pharmaceutical Benefits Agency	Makes direct decisions concerning dental and pharmaceutical benefits. Conducts cost-efficiency analyses to determine whether a pharmaceutical product qualifies for government subsidies	1
Public Health Agency	Has a national responsibility to promote good public health and ensure that the population is protected against communicable diseases. Provides soft law recommendations concerning public health issues	1
Health and Social Care Inspectorate	Conducts supervision to ensure that health and social care is safe, of good quality and provided in compliance with laws and regulations	2
Swedish Agency for Health and Care Services Analysis	Is a freestanding analysis agency and makes recommendations on a system level. Analyses health and social care services from the perspective of patients and citizens and reviews the outcomes of governmental activities	1
Swedish eHealth Agency	Leads and coordinates government e-health activities, such as electronic prescriptions of pharmaceuticals, to enable better information-sharing within health and social care	0
Swedish Association of Local Authorities and Regions (SALAR)	SALAR is an organization that represents and advocates regional and municipal government. Its relevance for national-level governance of de-implementation of LVC is in its leading role in the nationally coordinated organization for regional knowledge governance that was formed in 2016 and is still under development. The so-called national collaboration for knowledge governance relies on regional representation and responsibility for several national programme organizations and leads the way in knowledge governance for different areas of professional expertise	2

All stakeholders identified as potentially relevant participated in the study except for one government agency that declined participation because they did not perceive themselves as having any role in governing de-implementation. To recruit key informants for every stakeholder we used a snowball sampling strategy in which initial key individuals were identified and through which other informants were recruited [23]. For the initial contact with respective stakeholders, we searched for persons who had a formal role and experience related to the governance of the de-implementation of LVC. The identified persons mainly held various management positions including director-general and communication manager. Either the contacted persons were confirmed as being the right persons to include in the study, or they suggested other individuals who would be suitable based on their formal role, experience and knowledge of the issues under study.

In total, 12 informants were included in the study. One person declined participation due to time constraints, while only one informant from that stakeholder was interviewed. The number of informants from each stakeholder are listed in Table 1. The informants had different roles within the stakeholder organizations, being public servants in managerial positions (*n* = 9) or the director-general (*n* = 3). Seven (58%) of the informants were female.

All informants were contacted by email and asked to participate in the study. Before participating in the interview, they were informed both orally and in writing about the study purpose and procedures and were given the opportunity to ask questions. All informants received information that participation was voluntary, and all signed a written informed consent upon participation.

### Data collection

The data were collected through semi-structured interviews using an interview guide, which included open-ended questions about how the informant perceived the role of their own organization in the governance of the de-implementation of LVC as well as how they perceived the roles of other significant stakeholders. The interview guide also contained questions concerning the potential challenges involved in governing de-implementation. The interview guide was piloted in an interview at one of the participating stakeholder organizations, after which minor refinements were made. The pilot interview was excluded from the data analysis. The final interview guide can be found in Additional file 2.

The interviews were conducted between March 2020 and September 2021. They were conducted over the phone (*n* = 5), person-to-person at the informants’ workplaces (*n* = 2) and using Zoom (*n* = 5). Two interviews were conducted with two informants representing the same stakeholder, but all other interviews were individual. The purpose was to provide a broad and comprehensive understanding of the national-level governance of de-implementation in Sweden by focusing on the activities and challenges as described from the perspective of stakeholders with different roles in healthcare governance. As the study has an explorative standpoint and includes stakeholders with different governance roles, we did not expect to attain full data saturation for all identified themes, as they were expected to describe different activities to govern de-implementation based on their specific roles. Interviews were conducted by a researcher (HW) who has a PhD in medical sciences and who is also a licensed psychologist, or a research assistant experienced in business studies (BCM). Both were trained and experienced in interview techniques and had previous experience of interviewing healthcare actors. All interviews were audio-recorded using a digital recorder and transcribed verbatim. The interviews lasted an average of 53 (47–63) minutes.

### Data analysis

An inductive approach was applied, where the participants’ statements were openly coded through a thematic step-by-step analysis [19]. During the first step, the interview transcripts were read several times by one of the authors (BCM) to become familiar with the data corpus. At this stage, rough notes and early impressions were written down. In the second step, data fragments that highlighted something relevant to answer the research questions were coded using NVivo software (v.12 Pro). The process followed open coding (i.e., not having previous set codes), where codes were developed and changed throughout the coding process. Then, another author (HA) reviewed and analysed the codes separately. BCM and HA discussed the coding and agreed on a coding system.

In the next step of the analysis, the codes were screened for topics or issues that could be used as a centralizing, organizing concept for a theme. Both authors scanned the codes and the data within them, searching for everything relevant to address the research questions. As both authors worked through this joint analysis, new codes were generated, and existing codes sometimes changed. Codes were grouped into initial themes and subthemes. When this process was completed, possible differences between authors in how codes were categorized were discussed before moving to the next step. The data within each theme and subtheme were reviewed to assess whether they represented their codes and all relevant data while being distinct from each other. A theme was defined as something that had a certain pattern or meaning in relation to the research questions in the data. During the last step of data analysis, final themes and subthemes were defined.

## Results

The results section is divided into two parts: key stakeholders’ activities to govern de-implementation of LVC, and challenges involved in governing the de-implementation of LVC.

### Key stakeholders’ activities used to govern the de-implementation of LVC

The informants provided information about activities that had been used in the past, were currently used or could be used to govern de-implementation of LVC by any of the stakeholders. The results do not include any account of the frequency or duration with which these activities were used. The national-level governance of the de-implementation of LVC in Sweden can be distinguished by four potential activities: (1) recommendations, (2) health technology assessment (HTA), (3) control over pharmaceutical products and (4) the national system for knowledge management. Each of these themes and their subthemes are outlined in Table 2 and described in the text below.

**Table 2:** Overview of the themes and subthemes of activities used to govern the de-implementation of LVC at a national level and the stakeholders describing being involved in the activities

Theme	Subtheme	Stakeholders describing being involved in the activity*
Recommendations	Do-not-do recommendations	NBHW, Medical Products Agency
	Ranking of the priority of recommendations	NBHW
	Removal of outdated recommendations	NBHW, Medical Products Agency
	Recommendations after inspections	Health and Social Care Inspectorate
	Priority support	Swedish Agency for Health Technology Assessment and Assessment of Social Services
Health technology assessment		Swedish Agency for Health Technology Assessment and Assessment of Social Services
Control over pharmaceutical products	Withdrawal of pharmaceutical products	Medical Products agency
	Removal of the substitution of medicines	Dental and Pharmaceutical Benefits Agency
	Denial of price increase requests	Dental and Pharmaceutical Benefits Agency
National system for knowledge management		SALAR

*Not all included stakeholders are represented in the list of activities, as the representatives for some stakeholders did not report any current activities to govern de-implementation of LVC

#### Recommendations

Five different ways of using recommendations for governing de-implementation were described: do-not-do recommendations, ranking of the priority of recommendations, removal of outdated recommendations, recommendations after inspections, and priority support.

One of the described ways to govern the de-implementation of LVC was to provide recommendations in the national guidelines, developed by the National Board of Health and Welfare (NBHW), for methods that should not be used. These methods are labelled “do-not-do” and include methods that should be de-implemented because of their lack of scientific evidence and/or insufficient cost-effectiveness. The Medical Products Agency also produced treatment guidelines that could recommend against the use of a certain product for a specific condition or patient group. The national guidelines furthermore include information regarding the priority of recommendations, on a scale from 1 (*highest priority*) to 10 (*lowest priority*) based on the severity of the condition, the efficacy/effectiveness and the cost-effectiveness of the method. A ranking of 10 would imply that a method should not be widely used although it may not be formally labelled as do-not-do. The removal of outdated and no longer valid recommendations from the national guidelines was also considered as a strategy to govern de-implementation of LVC.

*We have something called do-not do recommendations, and that is our signal from the guidelines where we say that this is so bad that you should simply stop doing it*. (Informant 3)

The use of practices recommended against could also be noted and recommended against by the Health and Social Care Inspectorate when their inspections showed that an LVC practice had been used. However, assessing the use of LVC was not a systematic part of the agency’s inspections and only focused on practices that could cause harm to patients rather than LVC in general.

Another type of recommendation was developed in direct response to a government assignment to identify LVC practices in use and provide recommendations for their de-implementation. This assignment resulted in recommendations, called priority support, for de-implementation of three specific LVC practices (i.e., arthroscopic surgery for osteoarthritis of the knee, changing peripheral venous catheter at predetermined time intervals, corticosteroid injections for lateral epicondylitis). However, this was not a typical activity in governing de-implementation, and it was not continued beyond the specific assignment.

*Then we got a government assignment, and it was basically just that we would, I do not remember the exact writing, but that we should look at this with de-implementation of LVC, which was very surprising, because we should not give guidelines or develop guidelines or give recommendations, we should just say that this is what the scientific basis looks like. Then others get to take over. And this was really to point with the whole hand, this is a method that should not be used*. (Informant 2)

#### HTA

The HTA was another activity described to govern the de-implementation of LVC that was provided by the Swedish Agency for Health Technology Assessment and Assessment of Social Services. HTA entails reviewing both the effectiveness and potential side effects of a method by conducting meta-analyses of existing research evidence. The results are presented in HTA reports and used for decision-making as well as for informing national guidelines. Consequently, HTA results showing a lack of evidence for a method could potentially result in a recommendation to reduce or cease the use of this method.

#### Control over pharmaceutical products

This activity only applied to de-implementation of pharmaceutical products and medical devices, encompassing control of the safety of these products. Three different ways of using this activity for de-implementing LVC were described: withdrawal of pharmaceutical products, removal of the substitution of medicines and denial of requests for price increases. These activities were performed by the Medical Products Agency and the Dental and Pharmaceutical Benefits Agency.

Withdrawal of pharmaceutical products was used when a medicine or medical device was found to be unsafe, implying a clear de-implementation of the product. A more indirect way of using this activity was to reconsider and remove the substitution of medicines. This strategy could be used if the evidence for the medicine’s effectiveness was not convincing, if it was not cost-effective or if there were other better alternatives to use. Furthermore, requests from pharmaceutical companies for price increases of a product could be denied if other, better alternatives were available, which sometimes resulted in the companies choosing to withdraw the product from the market themselves. Thus, these activities implied the de-implementation of products based on cost-effectiveness issues.

*So, both price increases and reconsiderations are tools that we have that could contribute to more, at least indirectly, de-implementation, even if it is not the purpose. We do not initiate cases that, hey, now we are going to remove all old, junk drugs, we do not really do that. But it can be a consequence of different processes*. (Informant 6)

#### National system for knowledge management

A national system for knowledge management was described as having potential for governing the de-implementation of LVC at a national level. It had, however, not yet been used specifically for de-implementation purposes. The national system for knowledge management is a new initiative where one of the stakeholders (SALAR) has a role in supporting and coordinating the 21 regions’ knowledge management. This coordinated national system aims to support the use of more knowledge-based, equal and resource-efficient healthcare. It consists of different national programme areas where workgroups work to identify existing best evidence and potential research gaps while trying to support the implementation of best available evidence. Implementation of evidence was believed to have the indirect effect of also reducing the use of LVC. This shared system for knowledge was described as having the potential for wider, national dissemination of research evidence and thereby decreasing the risk of local practice variations or local uses of LVC practices.

*In this joint work, it can be said that we, we do not work specifically with de-implementation of LVC, but we rather work with identifying what is knowledge-based care*. (Informant 7)

### Challenges involved in the governance of the de-implementation of LVC

Three main themes of challenges were involved in the national governance of de-implementing LVC: unfavourable change incentives, ambiguous evidence and unclear roles. Each of these themes and their subthemes are outlined in Table 3 and described in the text below.

**Table 3:** Overview of the themes and subthemes of challenges involved in governing the de-implementation of LVC

Theme	Subtheme
Unfavourable change incentives	Vested interests
	Low priority for de-implementation of LVC
Ambiguous evidence	Insufficient evidence
	Quickly evolving evidence
Unclear roles	Decentralized decision-making
	No formal mandate to govern de-implementation
	Overlap of stakeholders’ functions and responsibilities

#### Unfavourable change incentives

This theme included two subthemes and concerned the challenges of de-implementation being a difficult and occasionally sensitive issue because of vested interests as well as having perceived low priority compared with the implementation of new evidence.

##### Vested interests

One reason why governing the de-implementation of LVC was considered challenging was the potentially vested interests in the methods being considered LVC. For instance, LVC could be an important part of a healthcare system, an organization or professionals’ routine practice, which may limit the incentives to de-implement the method as well as making it a potentially sensitive issue to work with from a governance perspective. One of the stakeholders described a project concerning de-implementation where the agency had produced recommendations advising against specific LVC practices. One of these recommendations (de-implementation of arthroscopic surgery for osteoarthritis of the knee) had stirred up emotions in the profession and been extensively criticized, which had garnered interest from the public media. Consequently, de-implementation was perceived as a highly challenging task.

*It was among the hardest things I have done here actually, to work with that. It felt like it took up all my time, for quite some time, and it says a lot if you go out and say, this is not something you should do anymore, it creates so much emotion, it is extremely difficult with de-implementation. Extremely difficult because there are so many who cling to their methods for various reasons*. (Informant 2)

Attempts to continue the identification and recommendations of reducing specific LVC practices were made after the publication of the first recommendations. However, due to previous criticism, it was perceived to be crucial that there was no ambiguity concerning the lack of effectiveness, which resulted in no methods fulfilling the criteria for being recommended against.

##### Low priority of de-implementation of LVC

The stakeholders noted that the expectations for implementing new evidence were much higher than those for de-implementing LVC practices. They considered getting new evidence into practice to be the main priority from both a governance and practice perspective.

*But we are clearly much more focused on the new, and what to do, rather than what not to do, although there is a part of it, of course, in this work, but it is not our focus….* (Informant 5)

The preference for new evidence was also reflected in the recommendations and guidelines, which were more focused on what to do than what not to do. The stakeholders emphasized the need for clearer recommendations on methods that should be de-implemented. It was also suggested that all do-not-do recommendations should be compiled in a specific do-not-do list to be published along with recommendations about evidence-based methods for use in the various national guidelines.

Although the stakeholders advocated for a more joint discussion concerning implementation of evidence-based practices and de-implementation of LVC practices, there was a belief among the stakeholders that implementation of evidence-based practices would automatically result in de-implementation of less effective practices: *I believe that the mechanism for de-implementation is really implementation* (Informant 8).

An exception to this was that if a pharmaceutical product had been shown to be harmful, then clear recommendations against the product were issued.

#### Ambiguous evidence

Ambiguous evidence encompassed two subthemes describing the challenges of not having clear evidence to base decisions on and recommendations about de-implementation of LVC.

##### Insufficient evidence

Insufficient evidence concerning the effectiveness of methods was described as an obstacle to providing clear recommendations for the use or nonuse of methods. The lack of evidence was often attributed to a shortage of studies, which made it difficult to distinguish between methods that are effective but have not been extensively evaluated and methods that are not effective and should be de-implemented. Therefore, it was often easier to state that a method lacked evidence rather than that it lacked effectiveness, which resulted in few methods being labelled as LVC.

*We have a very hard time showing that something is not effective. We can show that there is not much evidence that something is effective, but on the other hand, to say that we now know that it is not effective is difficult. We have only found a few areas where this is the case. Then you can implicitly conclude that if something has very poor evidence, it does not seem to have much effect based on the small evidence that exists and is also quite expensive. In that case we do not say stop using this, but we say those things and then the recipient must think for oneself that this is not a good thing*. (Informant 1)

Government agencies did not always interpret evidence consistently, which made it difficult to determine whether the evidence was unambiguous or clear enough to provide the basis for deciding whether a method should be labelled as LVC. One way to handle a lack of research evidence when developing guidelines was to include recommendations about further research and development concerning certain methods before they could be recommended for use or de-implementation. A scarcity of evidence was especially prevalent in some areas, such as social services. Consequently, there is a lack of guidelines and recommendations for both evidence-based methods within social services that should be used and LVC that should be de-implemented.

Another challenge for developing distinct de-implementation recommendations was the fact that guidelines are based on evidence from aggregated data and on averages which apply to groups of patients. Consequently, there will always be individual patients for whom the guidelines do not apply.

##### Quickly evolving evidence

The rapid development of evidence was another challenge for governing the de-implementation of LVC. The development of guidelines is time-consuming and will always lag behind current research. Thus, when guideline development is completed, the evidence situation may already have changed. This was considered especially challenging in some clinical areas, such as oncology, where the evidence for a method might change quite rapidly.

The progress of research was also described as a challenge for the healthcare professionals who must continuously keep up with the changing evidence. The stakeholders argued that there is a risk of information overload as professionals are expected to be up to date and knowledgeable about the latest evidence. Organizational structures or systems were requested to ease the burden for individual healthcare professionals to remain up to date.

*We all realize that knowledge changes fast. We need to create structures so that it is not individuals who need to carry it. It must be a system to make use of all knowledge*. (Informant 4)

#### Unclear roles

This theme included three subthemes describing the unclear roles among different levels in the system as well as within and between the national government agencies concerning their roles in governing the de-implementation of LVC.

##### Decentralized decision-making

The decentralized decision that made the Swedish system, with its 21 regions, represents a challenge for national government agencies in governing de-implementation of LVC. For a method to be widely de-implemented, all regions must agree that the method in question is of low value and decide to de-implement it. This requires continuous knowledge exchange and dialogue among the country’s 21 regions, which is difficult to establish and maintain. Furthermore, many decisions to use or de-implement a practice are up to the individual healthcare professional. Although the decentralized decision-making was perceived as a challenge, it was also described as beneficial in terms of adapting national guidelines to the local context to make them more easily applicable. In addition, national guidelines are not always available, and the regions may need to develop their own guidelines.

##### No formal mandate to govern de-implementation

There was a lack of clear governance for the de-implementation of LVC at a national level. The stakeholders perceived that they had an indirect responsibility to govern de-implementation since their responsibilities included supporting good care, using medicines rationally, providing evidence-based healthcare and striving to improve public health. However, they did not perceive that their own agency had a formal mandate to govern the de-implementation of LVC. This was the case for both stakeholders whose role was to provide recommendations, such as the NBHW, and stakeholders that made more direct decisions concerning the use of medical products, such as the Dental and Pharmaceutical Benefits Agency and the Medical Products Agency.

However, some differences in the means to act existed between stakeholders. Representatives from stakeholders providing recommendations noted that one reason for the lack of mandate was that they did not have the authority to decide how the regions should govern healthcare. Rather, they provided recommendations and priority support regarding which methods should be used and which should not, but the decisions and actual de-implementation of the methods were up to the regions. In contrast, the Medical Products Agency and Dental and Pharmaceutical Benefits Agency could use stronger actions such as withdrawal of products or removal of the substitution of medicines. The lack of formal mandates further implied limited time and resources to allocate to the de-implementation of LVC.

*We should contribute to a rational use of medicines and promote public health, so you can indirectly say, but it is not explicit as far as I know, [that we have a] mandate or mission to look at what should not be done*. (Informant 5)

##### Overlap of stakeholders’ functions and responsibilities

Some potential overlaps existed among stakeholders in governing the de-implementation of LVC, but conflicting perspectives or goals were considered quite uncommon. Overlaps of work implied a risk that the stakeholders communicated contradicting messages concerning the de-implementation of LVC. To mitigate this, they had dialogues about their ongoing work in cases where other stakeholders might have similar ambitions.

Overlaps between the national and regional levels of governance of the de-implementation of LVC were perceived to be more common. This included overlaps between national and regional HTA work and the development of guidelines. The recently adopted national system for knowledge-based management which coordinates the regions’ work on knowledge management was believed to have the potential to decrease the risk of overlaps and contradicting messages.

*Some years ago, all the county council directors decided to join forces and create a new knowledge management organization, and instead of doing it at the county council or regional level, we should gather and do everything together, so to speak, to avoid duplication*. (Informant 2)

## Discussion

The findings from this study show that national-level governance of the de-implementation of LVC in Sweden is conducted through four different activities: recommendations, HTA, control over pharmaceutical products, and a national system for knowledge management. However, the results also highlight that the national-level governance of LVC is limited. Although the interviewed stakeholders described various activities that could be used to govern de-implementation, they also explained that several of these were not used or only occasionally used in practice. The extent to which these stakeholders conducted activities to govern de-implementation differed, and representatives from two of the stakeholders did not report any current activities directed towards de-implementation of LVC. We identified challenges involved in governing the de-implementation of LVC in terms of various vested interests that result in the maintenance of LVC practices and low overall priority given to working with issues concerning the de-implementation of LVC compared with the implementation of new evidence. Ambiguous evidence made it difficult to clearly determine that a practice was LVC. Unclear roles, where no representatives for the stakeholders perceived that they had a formal mandate to govern the de-implementation of LVC, further contributed to the challenges involved in governing de-implementation of LVC at the national level in Sweden.

The highly decentralized healthcare system in Sweden, in which regions have considerable autonomy in the provision of healthcare, may provide a partial explanation for the limited national governance of the de-implementation of LVC. However, decentralization does not appear to hinder national stakeholders from involvement in governing implementation. Thus, other explanations for the limited governance of the de-implementation of LVC are needed.

Most of the described activities involved in governing de-implementation constituted soft governance [14] since they focused mainly on supporting de-implementation through information, knowledge management and nonbinding recommendations. One exception was de-implementation of pharmaceuticals, an area where stricter, harder governance was used, such as withdrawal of products. As such, the possibility for governing de-implementation of LVC differed among the included stakeholders. For instance, the NBHW was limited to making nonbinding recommendations about practices that should not be used, while the Medical Products Agency and the Dental and Pharmaceutical Benefits Agency could apply direct measures to restrict the use of products. Despite this, they reported similar challenges for governing de-implementation of LVC.

Effective health governance has been suggested to have three main functions: priority-setting, performance-monitoring and accountability arrangements [15]. Of these, only priority-setting activities were found to be used in the current study. Priority-setting was performed by supporting evidence-based decision-making through HTA and recommendations. It was also conducted by accounting for cost-effectiveness evaluations in prescribing through reimbursement decisions and recommendations for pharmaceutical benefits [15]. This indicates that there may be missed governance opportunities for reducing LVC in Sweden and that by increasing activities and improving systems for performance-monitoring and accountability, the governance of LVC could be improved. Performance-monitoring (e.g., feedback, public reporting, support for clinical decisions, and financial incentive) could be particularly interesting, as it has been suggested as a means to reduce LVC [24, 25]. One possibility for performance-monitoring at the national level is through the Swedish system of more than 100 national quality registries that provide an opportunity to monitor quality and the outcomes of healthcare [26]. To reduce the use of LVC, it is important to understand the factors influencing LVC and to tailor governance strategies to target these factors [11]. A recent study explored key national-level factors that promoted LVC use across three countries (the Netherlands, the United States and Canada) [9]. Our findings of national-level activities used to govern the de-implementation of LVC in Sweden targeted two of these key factors: the pharmaceutical and medical device industry and biased knowledge of care. Verkerk et al. [9] describe industry as a powerful actor contributing to LVC by advertising medical products to the population and through their influence on policy-makers and education and research funding. Our results showed that the control of pharmaceutical products, including the withdrawal of unsafe pharmaceutical products, removal of the substitution of medicines and controlling the pricing of products, was used for governing the de-implementation of LVC. Thus, there were some attempts to control the pharmaceutical and medical device industry. However, these activities did not fully address the industry’s influence on the public, policy-makers and research. The issue of biased knowledge was indirectly targeted by conducting HTAs and using information from these reports to inform national guidelines where recommendations of practices that should not be used are included. The other identified key factors, namely the payment system, fear of malpractice litigation, medical education and a “more is better” culture [9], were not directly targeted by the national-level activities.

Our study identified several challenges involved in governing the de-implementation of LVC in Sweden. In line with previous research [28, 29], we found that there are potentially vested interests in the methods considered to be LVC, which could make governing de-implementation a sensitive issue for national public agencies. Removal of an established practice entails a loss of autonomy for clinicians [30] and may be perceived as a critique and a lack of trust in clinicians’ expertise. It can be difficult for clinicians to accept that a practice they have provided to patients has been shown to be less than optimal [30, 31]. Furthermore, clinicians may perceive a practice to be integral to their professional practice and identity. In the current study, the sensitive nature seemed to create hesitation among the stakeholders to address the issue of governing the de-implementation of LVC. This highlights the fact that these types of deliberations and decisions may be highly complex, but they need to be made explicit and addressed for effective de-implementation [28].

One way for stakeholders to deal with the sensitive nature of de-implementation was to confine their recommendations for discontinuing or limiting the use of a method to only those cases when evidence concerning the lack of benefits for the method in question was very clear and convincing. Convincing evidence may help to overcome stakeholder resistance to de-implementation [32]. In fact, it has been suggested that de-implementation recommendations require more evidence than that needed to recommend the implementation of new methods, since evidence for the lack of effects and for the lack of harm is required when removing a practice [33]. However, providing evidence for the lack of benefits from established methods, rather than lack of evidence of benefits, has been shown to be inherently difficult from a methodological perspective [34]. In addition, there is limited research on assessing the evidence of already established methods [27, 32]. At the same time, the governance of de-implementation is challenged not only by a lack of sufficient evidence but also by the rapid development of evidence and information overload concerning new methods [35].

In line with previous research, our findings indicate that the governance of de-implementation receives lower priority than the governance of implementation for new and emerging practices [28, 36]. The implicit assumption seems to be that LVC will automatically be discontinued when there is evidence for new methods that supersede previous practices. This may be the case for some methods, but this reasoning has been criticized as an overly passive strategy that may not represent a sound policy approach to achieving excellence in healthcare [28]. Many studies report continued use of methods long after they have been identified as LVC and recommended against [37]. Clearly, the assumption or reasoning that de-implementation will automatically follow from implementation of new evidence has limited empirical support.

Recent years have seen a wider recognition of the importance of de-implementing LVC to achieve evidence-based healthcare, with increased attention paid to governance efforts to reduce LVC. One example is the increased focus on health technology reassessment (HTRA), in addition to HTA [36]. HTRA is defined as “a structured, evidence-based assessment of the clinical, social, ethical, and economic effects of a technology currently used in the healthcare system, to inform the optimal use of that technology in comparison with alternatives” [38]. Although the stakeholders in our study described some efforts to work with HTRA with the objective of identifying practices that should be de-implemented, it is not yet a consistent activity in the national governance of the de-implementation of LVC in Sweden.

## Implications for research and practice

An evidence-based healthcare system requires both implementation of new evidence and de-implementation of LVC. The relatively low priority of de-implementation of LVC from a national governance perspective in Sweden implies that a potentially important opportunity to create conditions for evidence-based healthcare is not being used. Our findings add to the body of literature showing an emphasis on implementing new evidence and the implicit assumption that de-implementation will happen automatically. This implies an obvious risk for continued LVC use alongside evidence-based practices. Further discussion about implementation *and* de-implementation in all the mentioned activities (recommendations, HTA, control over pharmaceutical products, and national system for knowledge management) may be one way forward to achieve an improved understanding of the challenges of de-implementation. This includes an increased focus on HTRA, incorporating do-not-do recommendations in the national guidelines, and developing a cohesive national system for knowledge management including de-implementation of LVC. Governance activities were mainly related to priority-setting, which indicates that there may be missed governance opportunities to reduce LVC by supporting systems for performance-monitoring and accountability that address LVC.

Addressing the challenges identified in this study could facilitate a more systematic governance of de-implementation at a national level. The challenges of de-implementation as a sensitive and difficult issue together with the challenges introduced by the decentralized healthcare system imply a need to create transparency in how and on what grounds de-implementation recommendations are made and a collaboration between the different levels in the healthcare system (i.e., national, regional and local). Specifically who has what responsibility and mandate regarding the de-implementation of LVC must be clear. The national system for knowledge management has the potential to facilitate this. However, this system is currently under development, and our findings indicate that the focus is mainly on coordination of the implementation of new evidence, and little attention is given to the de-implementation of LVC.

## Limitations

Twelve interviews were conducted in this study, which may be considered a relatively small sample. However, the stakeholder representatives provided highly relevant information for the study; that is, the information power was high. Research suggests that the more information power a sample holds, the fewer the interviews that are needed [39]. According to Guest et al. [40], 12 interviewees should be sufficient if the informants are knowledgeable about the subject, data quality is satisfactory, and the aim is to understand common perceptions and experiences rather than to assess variation between groups. All stakeholders identified as potentially relevant participated in the study except for one government agency that declined participation because they did not perceive that they had a role in governing de-implementation. It should be noted that the purpose was to include all relevant stakeholders and investigate their roles and perspectives on the de-implementation of LVC to obtain a broader and deeper understanding of the topic rather than to achieve data saturation. Based on the stakeholders’ specific roles, they described different activities to govern de-implementation of LVC. Despite these differences, the informants perceived similar challenges in governing de-implementation of LVC, although all informants did not mention all challenges.

The findings represent activities that the stakeholder representatives described as activities that have been used previously, are currently used or may be used in the future to govern de-implementation, either by the stakeholder from which the representative came or by any of the included stakeholders. We have limited knowledge of when and to what extent the described activities are or were used. It is possible that the descriptions by the representatives give a sense of more extensive work on governing de-implementation than is the actual case. However, it is also possible that they neglected to describe some activities. We did not investigate to what extent the activities had been acknowledged by healthcare providers to steer or impact the de-implementation of LVC. It is also possible that other stakeholders besides national government agencies—such as patient and professional associations—have an indirect influence on the governance of de-implementation, but these were not addressed in this study. Considering Sweden’s administrative model of independent public agencies and the decentralized health and welfare system, more research is needed to account for complementary perspectives and data on LVC-related activities and challenges at other levels of healthcare. Investigation of the regional governance of de-implementation of LVC is particularly important, and we are planning a future study on this.

This study investigated the governance of de-implementation in Sweden. Consequently, the study findings cannot be directly transferred to other healthcare systems. Nevertheless, we believe that many of the identified challenges apply also to other countries, which is supported by previous research that has identified overlapping challenges [28] and key factors shared across different healthcare systems [9]. We have also attempted to enhance transferability by including a thorough description of the research context.

## Conclusions

Evidence-based healthcare requires implementation of evidence-based methods as well as the de-implementation of LVC practices. Although various activities are performed to govern the de-implementation of LVC in Sweden, these are limited and have lower priority than the implementation of new methods. Challenges to the governance of de-implementing LVC relate to the unfavourable change incentives involved, an ambiguous evidence base, and unclear roles and mandate to govern the de-implementation of LVC. Addressing these challenges could create a more systematic governance of de-implementation at a national level and thereby create conditions for reducing LVC in healthcare.

## Supplementary Information

**Additional file 1.** The Standards for Reporting Qualitative Research.

**Additional file 2. **Interview guide.

## Data Availability

The datasets generated and/or analysed during the current study are not publicly available due to integrity of participants but are available from the corresponding author on reasonable request.

## Abbreviations

HTA: Health technology assessment
HTRA: Health technology reassessment
LVC: Low-value care
NBHW: National Board of Health and Welfare
