# Supplementary material for: National governance of de-implementation of low-value care: a qualitative study in Sweden
Source: Health Res Policy Syst. 2022 Sep 1;20:92. doi: 10.1186/s12961-022-00895-2 (PMC9438133; doi:10.1186/s12961-022-00895-2)
Supplement: Supplementary file 2 — Additional file 2. Interview guide. [file 12961_2022_895_MOESM2_ESM.docx]

**Additional file 2**. Interview Guide

**Interview Guide**

**Some key concepts:**

De-implementation: with the meaning of reducing or completely ceasing the use

Ineffective care (in the first place), low-value care (also works well)

Governance: national (state) and regional stakeholders who act (in different ways) in order to influence how care is conducted

**Introduction**

“This interview will be about what role you (the agency's name) have in governing de-implementation of ineffective care. By ineffective care I mean care that has a low value for the patient and for the healthcare system, eg, because there is no evidence that the practice has a positive effect, or is even harmful, or that the costs are too high in relation to the benefits (ie, not cost-effective). By care we mean a wide range of interventions, from medicines to other types of treatment, but also tests and diagnostic methods.

In this study, we turn in a first step to stakeholders who have a role in the national governance of health care and thus can influence that elimination takes place—ie, that ineffective methods are reduced or completely stopped. The purpose of the study is to understand more about how national stakeholders view their own role and responsibility for governing de-implementation, as well as the roles and responsibilities of other stakeholders.”

**Background questions**

1. Can you briefly describe your role in (the agency´s name)?
2. What experience do you have from working with issues related to de-implementation?
3. When we talk about de-implementing ineffective care, what do you think is included in that concept? Is there anything in particular that you think about?

**The stakeholders’ work concerning governing de-implementation of ineffective care**

1. If you were to briefly describe how you (the agency´s name) work with issues concerning de-implementation of ineffective care - what does it look like? What do you do? What activities does it involve in concrete terms?

*"Within this study, we are interested in the governance of the de-implementation of ineffective care—ie, what national (state) and regional stakeholders do to encourage the elimination of ineffective care. Now I will ask a few questions concerning how you view the role and responsibility of different stakeholders for governing de-implementation. We will start at the national level, but I will also ask questions about what is done at the regional level and what role the profession has in de-implementation—in the meeting with the patient".*

**Perception of own role and responsibility: National level**

1. Based on the national level of governance, how would you describe (agency´s name)'s role in governing the de-implementation of ineffective care? What function do you fulfil?
2. Do you have any responsibility for the de-implementation of ineffective care?

**Perception of other stakeholders’ roles and responsibilities: National level**

*"There are also other stakeholders involved in governing healthcare at the national level."*

1. How do you perceive the role of other national stakeholders in making the de-implementation of ineffective care a reality? What function do they fulfil?
2. What do you think is their responsibility? What can be expected from them?
3. How do you think the governance of de-implementation of ineffective care at the national level works?
4. (Sampling Question) If we want to understand how the de-implementation of ineffective care is governed at the national level - which stakeholders do you think are the most important for us to include?

**Perception of other stakeholders’ roles and responsibilities: Regional level**

*"There are also stakeholders involved in governing healthcare at the regional level."*

1. How do you see the role of regional stakeholders in realising the de-implementation of ineffective care? What function do they fulfil?
2. What do you think is their responsibility? What can be expected from them?
3. How do you think the governance of de-implementation at the regional level works?

**Perception of the profession's autonomy**

*"If we switch to another level in the system and look at what happens in everyday healthcare practice, in the meeting between patient and professional":*

1. What role do you think the profession has in de-implementing ineffective care?
2. What do you think is their responsibility? What can be expected from them?
3. In the work related to governing de-implementation of ineffective care that (the agency’s name) do, how do you consider the profession's autonomy and the opportunity to make independent decisions in the meeting with the patient?

**Patient**

1. How do you view the patient's role in de-implementing ineffective care? Do patients have any responsibility?

**Bonus**

1. How do you see the governance of de-implementation of ineffective care in the future? Will there be any changes in terms of roles and responsibilities at the national and/or regional level?
2. What do you consider to be the biggest challenge in governing the de-implementation of ineffective care?
3. What do you think works particularly well in the governance of the de-implementation of ineffective care?
4. Would you like to add anything else?
5. (Sampling question) is there anyone else in your organization who has knowledge and experience with these issues that we should meet?
